# Single Cell Transcriptomics: Methods and Applications

**DOI:** 10.3389/fonc.2015.00053

**Published:** 2015-03-10

**Authors:** Itamar Kanter, Tomer Kalisky

**Affiliations:** ^1^Faculty of Engineering, Institute of Nanotechnology, Bar-Ilan University, Ramat Gan, Israel

**Keywords:** gene expression, single cell, qPCR, RNA sequencing, FISH

## Abstract

Traditionally, gene expression measurements were performed on “bulk” samples containing populations of thousands of cells. Recent advances in genomic technology have made it possible to measure gene expression in hundreds of individual cells at a time. As a result, cellular properties that were previously masked in “bulk” measurements can now be observed directly. In this review, we will survey emerging technologies for single cell transcriptomics, and describe how they are used to study complex disease such as cancer, as well as other biological phenomena such as tissue regeneration, embryonic development, and immune response.

## Introduction

Since the late 1980s, the invention of PCR (1), the microarray (2, 3), and more recently, next generation RNA sequencing (4) has provided enormous amounts of gene expression data. These technologies, along with the information revolution, have reshaped biology as a quantitative and computationally intensive science, and are expected to pave the way for personalized therapy of cancer, auto-immune disease, and other complex genetic conditions. An early demonstration of the power of high throughput genomics can be seen in the work of Sorlie et al. (5), who used gene expression microarrays and hierarchical clustering to group 85 breast tissues and tumors into subtypes based on their gene expression patterns. Each breast cancer subtype was found to have a distinct “molecular portrait,” prognosis, and recommended treatment. In another work, Whitfield et al. (6) used microarrays to measure gene expression in synchronized HeLa cells over time, and found >850 genes that were periodically expressed during the cell cycle. By hierarchically clustering gene expression patterns, they identified co-expressed groups of genes known to be involved in various cell cycle processes such as DNA replication and chromosome segregation, along with genes of previously uncharacterized function.

To provide sensitive and reliable measurements, microarrays typically require 1–2 μg of total RNA, which corresponds to a “bulk” of ~100,000 cells. This requirement has limited genomic studies to “whole tissue” measurements, providing an expression profile that was “averaged” over all the cells in that tissue. However, multicellular organisms consist of different cell types, each having a different role and a different corresponding transcriptional profile. Furthermore, even cells in more homogeneous systems such as a bacterial colony or a mammalian tissue culture vary according to intrinsic stochastic variation (7–9) and extrinsic variation governed by differences in the cell cycle stage or their local environment (10). Thus, “bulk” measurements often “average out” information that is critical to proper understanding of fundamental biological phenomena and complex disease.

In the last decade, rapid advances in genomics have led to the development of high throughput technologies that allow for hundreds – or even thousands of genes – to be measured simultaneously in hundreds of individual cells. To date, single cell transcriptomic technologies have been used to characterize rare cell populations like circulating tumor cells (11), cancer stem cells in solid tumors (12, 13), and embryonic stem cells (ESCs) in mammalian blastocysts (14). Furthermore, single cell technologies allowed direct measurement of gene expression variability originating from the stochastic nature of gene expression (7, 9, 15, 16) or from other more extrinsic sources such as the cell cycle (17) or the circadian rhythm (18).

In this manuscript, we will briefly review some of the latest developments in single cell transcriptomic analysis and their applications to biology and medicine.

## Methods for Single Cell Transcriptomics

**Figure 1:**
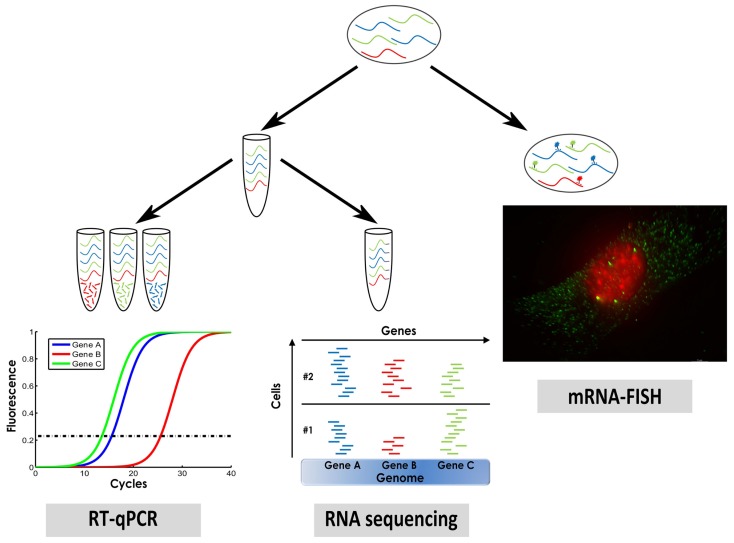
**A sketch of three methods for measuring single cell gene expression that were described in this manuscript: mRNA fluorescence *in situ* hybridization (mRNA–FISH), single cell qPCR, and single cell RNA sequencing**.

We will describe three methods that are widely used for measuring single cell gene expression: mRNA *in situ* hybridization, single cell quantitative PCR (qPCR), and single cell RNA sequencing (Figure 1).

### Single molecule mRNA fluorescence *in situ* hybridization allows to count single transcripts in individual cells within an intact tissue

Single molecule mRNA–FISH is a technology for fluorescently labeling and counting mRNA molecules in fixed cells or tissues. In the first step, probes are designed to target specific mRNA molecules. The probes are oligonucleotides that are covalently bound to fluorochromes and whose sequence complements the sequence of the target mRNA transcript. When the probes are mixed with a chemically fixed cell or tissue sample, they hybridize to the target mRNA molecules inside it. By proper image analysis, individual mRNA molecules can be visualized as fluorescent spots under a microscope and the number of transcripts can be automatically determined by counting (19). Multiple sets of spectrally separated fluorochromes can be combined in order to count two to three mRNA species (i.e., genes) simultaneously (20). The main limitation of mRNA–FISH is the relatively small number of genes than can be simultaneously measured. However, super-resolution microscopy can used to increase the detection capacity to 32 genes simultaneously (21), or even more by sequential rounds of hybridization and washing (22). Since mRNA–FISH is based on imaging, it also provides spatial information regarding the sub-cellular localization and distribution of the transcripts (23). For example, transcription sites can be identified as enlarged spots and the number of nascent mRNA molecules can be estimated (24). Furthermore, when implementing mRNA–FISH on tissues, it is possible to obtain single cell gene expression along with the original tissue microstructure (25) such as colon crypts (26) and nephrons (27).

### Microfluidic single cell qPCR is a sensitive tool for measuring the expression of multiple genes in hundreds of individual cells

Quantitative PCR is widely used to measure gene expression. Following cell lysis, RNA purification, and reverse transcription, copies of chosen transcripts – as defined by specific primers – are repeatedly replicated and their quantity is monitored over time by a fluorescent reporter dye. The primers are short oligonucleotides specifically designed to bind the target transcript at the 5′ and 3′ ends, thus enabling the DNA polymerase to initiate reverse transcription and replication. Since qPCR is based on amplification, it highly sensitive and can detect even single molecules (28). Single cell measurements typically require thousands of reactions per experiment (e.g., 100 cells × 100 genes = 10,000 reactions). In order to overcome this limitation, microfluidic single cell qPCR uses PDMS microfluidic chips with matrix-like architecture to combinatorially mix up to 96 individual cells and 96 primer pairs into 9,216 independent qPCR reactions on a single chip (29, 30). Single cell isolation is typically done by flow cytometry or micromanipulation (31). In contrast to “bulk” qPCR, in single cell qPCR no purification steps are possible due to the low amount of starting material (32). Thus, all steps following single cell sorting such as cell lysis, reverse transcription, and target transcript amplification must be performed sequentially in a single tube.

From our experience, the main limitation of single cell qPCR is the need to choose in advance which genes to measure – which limits our ability to discover novel biomarkers without some prior knowledge, and the fact that spatial information of the tissue structure is lost. On the other hand, those ~100 genes that are chosen can be measured in hundreds – or even thousands – of individual cells in a relatively high dynamic range (5–7 orders of magnitude for most genes), provided that the primers are chosen carefully to account for varying reverse transcription efficiencies.

### Sequencing all mRNA molecules in a single cell enables us to measure thousands of genes simultaneously and to discover novel biomarkers

The first steps in RNA sequencing are cell capture and lysis, followed by reverse transcription, whole transcriptome amplification, and next generation sequencing. The expression levels of a gene can be inferred from the number of sequencing reads that align to the genome in the location of that gene. A variety of priming methods exist, the majority of which use either random or poly-A primers. Likewise, different amplification schemes are available based on PCR or linear amplification. Recent developments include “barcoding” of individual samples (cells) with unique sequences in the priming step, which allows the barcoded cDNA to be pooled, amplified, and sequenced in a single sequencer run. Later, each read is *in silico* attributed to its original cell according to its barcode (33–37). Similar barcoding can be done on single transcripts allowing for direct counting of molecules (38–40).

A major challenge in the field of single cell genomics is to develop sensitive, precise, and reliable technologies for sequencing the whole transcriptome from hundreds – or even thousands – of individual cells. At present, RNA sequencing is still too expensive and work-intensive for high throughput single cell measurements. Furthermore, the amplification step introduces large measurement noise and uncharacterized bias, especially in low abundance transcripts (41). Two remarkable technologies are emerging for automated isolation, lysis, and whole transcriptome amplification from individual cells at high capacity. The first approach uses automated microfluidic chips (42, 43) with nano-liter chambers for 96 individual cells. The use of nano-liter volumes has been shown to result in more accurate measurements and decreased amplification bias when compared to tube-based preparations of single cells. The second approach uses droplet microfluidics (44) for ultra-fast (~1 kHz) isolation of single cells in separate sub pico-liter drops immersed in oil. Each drop is then further processed automatically through the next steps that include cell lysis, whole transcriptome amplification, and barcoding. The emulsion can then be broken, sequenced, and individual cells can later be de-multiplexed computationally according to their barcode.

## Applications of Single Cell Transcriptomics

### Characterization of the cell sub-population repertoire of tissues and tumors is essential for understanding how tissues regenerate and for designing new targeted therapies for cancer

Many tissues in the human body such as the skin, the inner lining of the intestine, and the blood and immune systems are constantly regenerating. These tissues are dynamic systems composed of different cell types including stem cells, progenitors, and differentiated cells of various phenotypes. The stem cells are long lived cells that maintain the regenerative potential of the tissue. Some of their progeny become progenitors, cells that rapidly divide and proliferate. These in turn differentiate into cells with more and more specialized functions. Turnover times range from 4 to 5 days in the intestinal epithelium (45) to a few months in the blood (46). Abnormalities in this process presumably result in tumors. For example, it was found that many tumors have a small sub-population of “cancer stem cells,” i.e., cells with tumorigenic potential that can regenerate a new tumor when transplanted into an immune-deficient mouse (12, 47, 48).

Since the stem cell population is a small minority (<1%), single cell analysis is required in order to identify, isolate, and molecularly characterize them. Transcriptional profiling of the various cell types in tissues and their corresponding tumors will allow for better understanding of the process of tissue regeneration and tumor formation. Furthermore, molecular characterization of the stem cell population will enable design of more efficient anti-cancer drugs that will specifically target the stem cells of the tumor while leaving all other cells viable.

### Example: regeneration in the intestinal epithelial crypt

**Figure 2:**
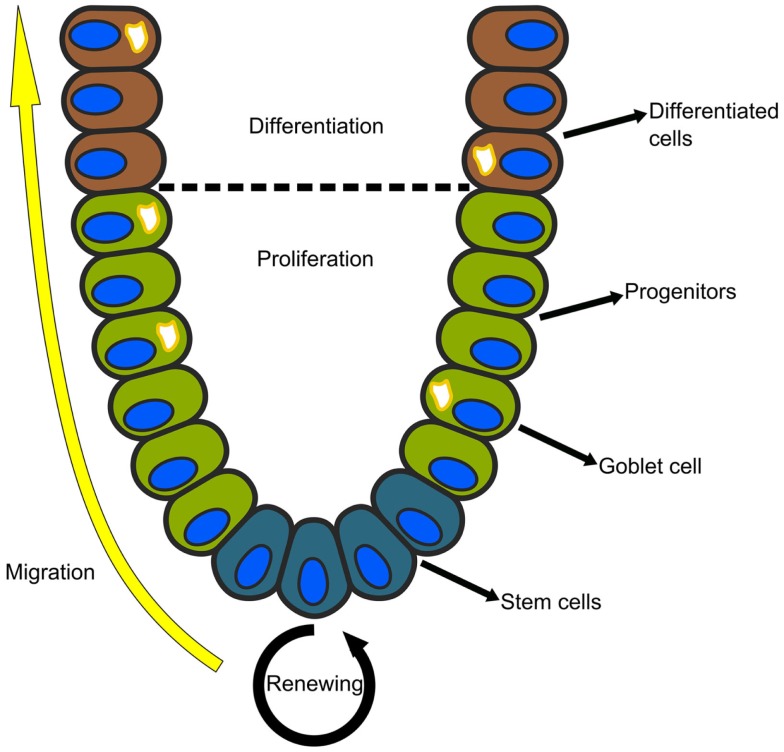
**Each crypt in the mammalian small intestine or colon is an independently regenerating unit**. The stem cells reside at the bottom and their progeny migrate upwards as they proliferate and differentiate. In the colon, there are two main cell lineages: absorptive enterocytes and secretory goblet cells.

The epithelial lining of the mammalian small intestine and colon is organized into glands called the crypts of Lieberkühn. Each crypt is a regenerating unit in which the stem cells reside at the bottom, and their progeny migrate upwards as they proliferate and differentiate (see Figure 2). In the colon, there are two main cell lineages: enterocytes, which reabsorb fluids and electrolytes, and goblet cells, which secrete protective mucus and other growth factors (45). Although studied for decades, the gene circuits that govern the regeneration of the intestinal crypt, as well as the distortions that lead to colon cancer, are not well understood.

In order to better characterize the repertoire of cell sub-populations in intestinal tissues and tumors, we used a combination of flow cytometry and microfluidic single cell qPCR to molecularly profile the cellular composition of primary human colon cancer epithelia (13), and showed that human colon cancer tissues contain distinct cell populations whose transcriptional identities mirror those of the different cellular lineages of normal colon. We also showed that single cells from human xenografts implanted in immune-deficient mice can regenerate a similar but distorted cell population repertoire of the parent tumor. In another work (49), we applied similar methods to the mouse colon epithelium and found a previously unknown sub-population of goblet cells that were interdigitated with the Lgr5(+) stem cells and which secrete growth factors that maintain stem cell homeostasis, similar to Paneth cells in the small intestine.

Although single cell qPCR can measure up to 96 genes simultaneously, it is limited by the fact that all information regarding tissue microstructure is lost. To overcome this, mRNA–FISH can be used to study spatio-temporal processes in tissue regeneration. Itzkovitz et al. (26) applied three color single molecule mRNA–FISH to follow the expression level of a set of putative stem cell markers in the intestinal crypt. They revealed that all the markers overlapped in the bottom of the crypt and co-expressed with Lgr5(+) cells. These results support the hypothesis of the existence of only one stem cell population residing at the crypt base. In a later study (50), the authors used a combination of lineage tracing and single molecule mRNA–FISH to study the design principles of crypt formation in the small intestine of infant mice, and found that in the first stages of development, there is a surge of symmetric stem cell divisions, which are later followed by a transition to asymmetric stem cell divisions as the crypts continued to grow.

### A detailed picture of the early stages of embryonic development emerges from transcriptional characterization at the single cell level

Embryogenesis involves complex growth and differentiation events by which new tissues and organs are formed. Subtle but essential differences in gene expression between seemingly homogeneous cells at early stages can have a dramatic long-term effect on their fate and that of the organism as a whole. Often, sharp boundaries are formed by direct interaction between adjacent cells (51). In order to understand the regulatory changes that govern this process, and in particular, the failsafe mechanisms that ensure normal embryogenesis under varying and “noisy” conditions (52), transcriptional profiling of individual cells is required, rather than whole embryos or whole tissues (53).

**Figure 3:**
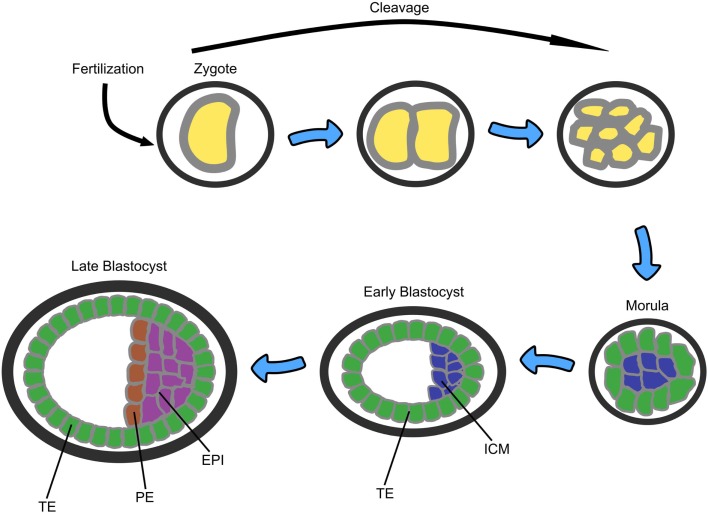
**A sketch of the early stages of mammalian embryonic development starting from zygote, through morula (8–16 cells), to blastocyst (32–64 cells)**. TE, trophectoderm; ICM, inner cell mass; PE, primitive endoderm; EPI, epiblast (53).

In early human embryogenesis following fertilization, the single cell zygote undergoes early cleavage divisions to form the morula – an apparently non-differentiated mass of 8–16 cells (see Figure 3). The morula then differentiates into two cells types: the outer layer, also called the trophoblast or trophectoderm (TE), which is responsible for implantation of the embryo into the wall of the maternal uterus and which will later develop into the placenta, and the inner cell mass (ICM), from which ESCs are derived. The resulting structure is called the blastocyst and consists of 32–64 cells. The ICM then further differentiates into two additional layers: the primitive endoderm (PE), which provides developmental cues to the embryo, and the epiblast (EPI), which will become the embryo itself (54).

In order characterize the transcriptional changes during this process, Kurimoto et al. (55) used single cell qPCR and whole transcriptome amplification followed by microarray analysis to measure single cell gene expression in a mouse blastocyst. They isolated ~20 single cells from the ICM, a population of undifferentiated pluripotent cells that give rise to all embryonic lineages and which is also the source of ESCs, and found that even in rather early stages of the blastocyst (E3.5, ~32 cells), where the cells are morphologically homogeneous, two populations of cells could be discerned: one with PE-like gene expression and the other with pluripotent EPI-like gene expression. The genes that were differentially expressed between these two populations were found to be preserved in the morphologically differentiated PE and EPI in the embryos 1 day later (E4.5).

Guo et al. (14, 53) used microfluidic single cell qPCR to measure the expression level of 48 genes in parallel from >500 individual cells from different stages of mouse pre-implantation development, from the 1-celled zygote through to the 64-celled blastocyst. They found that even at the 16-cell stage morula, the inversely correlated genes Id2 and Sox2 could discern between the inner part of the morula (Id2^low^/Sox2^high^), which further develops into the ICM, and the outer part that develops into the TE and that could be specifically stained by a cell-membrane labeling fluorescent dye prior to embryo dissociation. They also observed expression heterogeneity within the early ICM of the 32-cell blastocyst, for example, in the anti-correlated ligand–receptor pair Fgf4 and Frfr2. Later on at the 64-cell ICM, Fgf4 becomes restricted to the EPI and Fgfr2 to the PE.

Tang et al. (56) studied the process by which cells from the ICM, cultured *in vitro*, transform into ESCs and obtain infinite capacity for self-renewal and pluripotency. By performing single cell qPCR and single cell RNA sequencing at different time points, they were able to identify clusters of genes and microRNA’s whose expression increased or decreased during this process, as well as genes that gradually lost cell–cell heterogeneity (e.g., c-Myc) and genes whose splicing was modified. Xue et al. (57) used single cell RNA sequencing and weighted gene co-expression network analysis (WGCNA) to study cross correlations between genes during development from oocyte to morula in mammals. They were able to identify 25 distinct modules of co-expressed genes, each with a defined function. Nine of these modules were found to be stage specific, indicating that transcriptional changes occur in step-wise fashion throughout development. Furthermore, they found that these modules were similar in human and mouse, but diverged in their time of activation.

Similar works were done in other organisms. Recently, Hashimshony et al. (37) used single cell RNA sequencing to characterize lineage specific gene expression in the very first stages of development of a *C. elegans* embryo. By learning to identify differential gene expression among sister cells (blastomeres) during cleavage, they produced a classifier based on a small set of key informative genes that could distinguish between closely related sister cells. Later stages of development in specific tissues were also studied at the single cell level. For example, Treutlein et al. (58) used microfluidic single cell mRNA sequencing to follow the development of lung alveolar epithelial cells in a mouse embryo. Using unsupervised clustering, they were able to identify and characterize five epithelial cell types, four of which were associated with previously reported cell types. Based on the expression of specific marker genes, they reconstructed the differentiation pathway of progenitor cells into separate alveolar cell lineages.

### Single cell measurements can reveal new dimensions of heterogeneity as well as transcriptome-wide relations between genes

According to the central dogma of molecular biology, formulated by Francis Crick in 1970s (59), the transcription of genes from DNA to RNA is the first stage of information flow in the cell. However, it was recently realized that transcription is an inherently “noisy” process (15). Due to the relatively small number of molecules involved (DNA, RNA polymerase, etc.), even genetically identical cells will have different expression levels of the same gene (60). This “randomness” has an essential role in key cellular activities (61) as well as implications on gene expression measurements, whereby single cell measurements yield a more detailed and deeper understanding of many intra-cellular processes that were masked or “averaged out” by traditional “bulk” techniques. For instance, new cell states and cell types in tissues can be resolved by identifying groups of individual cells having distinct expression profiles (62). Furthermore, due to the inherent variability between cells, every cell for which we measure the expression of multiple genes is essentially a different “experiment” that reveals new information about the relations between different genes (63, 64). Thus, measuring the expression of multiple genes simultaneously in hundreds of individual cells can be used to identify pairwise correlations between them and to reconstruct transcription regulation networks (65). Comparison between these networks in normal and disease states can provide deeper mechanistic insight to the underlying effects of disease (66).

Gandhi et al. (67) explored the degree of gene expression coordination between genes in individual cells of *Saccharomyces cerevisiae*. They used multicolor FISH labels to measure expression levels of related and unrelated pairs of genes and found very high coordination between functionally related genes that were temporally induced by the sugar galactose (GAL). In contrast, other more constitutive genes – even those that were functionally related or that had identical promoters – were found to have low correlation to each other, most likely due to stochastic fluctuations independently affecting individual genes.

Shalek et al. (68) used single cell RNA sequencing and mRNA–FISH to provide a more accurate and detailed description to the response of a seemingly homogeneous population of cells to external stimuli. They examined the response of mouse bone-marrow-derived dendritic cells (BMDCs) to lipopolysaccharide (LPS), a toxin found in the outer membrane of Gram-negative bacteria that elicits a strong immune response, and found that 185 of the 241 most variable genes had bimodal expression patterns. They also found that many key genes involved in the immune response that are highly expressed at the population level actually exhibit a bimodal expression pattern at the single cell level. In addition, they found cell to cell heterogeneity in splicing patterns, whereby genes that had multiple splicing isoforms at the population level exhibited predominantly only one pattern in any specific individual cell.

Another dimension of heterogeneity was revealed by Hansen et al. (69), who studied allele-specific gene expression in single cells by using mRNA–FISH probes that were designed to distinguish between different alleles (paternal vs. maternal) of the same gene according to their single-nucleotide variants (SNVs). Using this method, allelic expression imbalance of the genes *Nanog* and *Chd4* was measured in a mouse ESC line. For example, they found that although under certain conditions (such as changing the growth medium to 2i medium – a medium with minimal requirements for self-renewal) the total number of expressed transcripts increased, the proportion of cells exhibiting mono-allelic expression remained the same. A more transcriptome-wide approach was taken by Deng et al. (70), who used single cell RNA sequencing to measure allele-specific expression in mouse pre-implantation embryos of mixed background. They showed that a large fraction (~25%) of all autosomal genes was expressed in mono-allelic fashion, which appeared to be stochastic and independent.

The effect of variations in the DNA on gene expression was further investigated by Weinstein et al. (71), who devised a method combining single cell qPCR and Sanger sequencing for simultaneously measuring gene expression profiles in hundreds of individual B-cells as well as the mutations in genes coding for their antibody heavy and light-chains. By measuring both expression and mutations in B-cells of mice following immunization, they were able to probe the relation between B-cell activation, proliferation, and differentiation and antibody mutation during “affinity maturation” – the process by which B-cells are stimulated to divide and hyper-mutate in order to increase their affinity for a specific antigen. A different aspect of the relation between the genome and transcriptome was investigated by Wills et al. (72), who used microfluidic single cell qPCR to associate single-nucleotide polymorphisms (SNPs) with gene expression phenotypes. By measuring the expression levels of specific genes from the Wnt pathway along with their associated SNPs in hundreds of single cells derived from 15 individuals, they were able to show that SNP’s have considerable effect on the expression level statistics of each individual. This feature can only be observed at the single cell level, and is “masked out” in bulk experiments where many cells are averaged.

### Measuring the spatial distribution of mRNA molecules within a cell uncovers fundamental mechanisms involved in cell motility and development

Sub-cellular localization of mRNA provides a mechanism to spatially control gene expression and protein distribution within the cell. Differential localization was observed in mRNA transcripts encoding cytoskeletal proteins (73) and embryonic morphogens (74). In a more recent study, Lécuyer et al. used mRNA–FISH to perform comprehensive analysis of mRNA localization in early *Drosophila* embryos (75). Their observations showed that a high fraction of genes – 71% out of 3,370 genes analyzed – were expressed in spatially distinct patterns. Furthermore, they found a strong correlation between mRNA distribution and subsequent protein localization and function. Taken together, transcript abundance measurements along with spatial information and patterning over many cells will provide a richer description of transcript properties as well as better understanding of the functional interactions between genes (23).

## Outlook

Single cell transcriptomic technologies are still developing and we believe that they have not yet reached their full potential. One intriguing development is the ability to perform transcriptomic measurements while preserving the tissue context and sub-cellular localizations, for example, by cryo-sectioning tissues into thin slices and sequencing the whole transcriptome in each slice independently (76, 77), or even by sequencing every single transcript in primary tissue under a microscope while maintaining spatial information as to the location of each transcript within each individual cell (78). Another challenge is to increase the number of single cells that can be analyzed: many thousands of cells are required to gain thorough understanding of complex biological processes such as cancer or the development of an embryo (62, 79). Similar capabilities have recently been developed to quantify proteins by combining mass spectrometry and flow cytometry (80). In the next few years, we expect all these novel technologies to provide a new wealth of data. Parallel progress will be required to develop computational tools to visualize and analyze this data and deduce testable hypotheses (81–85).

## Conflict of Interest Statement

Tomer Kalisky is co-inventor of a patent application licensed by Stanford University to Quanticel Pharmaceuticals Inc. and was awarded shares in Quanticel Pharmaceuticals Inc. as a result of this licensing agreement. Itamar Kanter declares that the research was conducted in the absence of any commercial or financial relationships that could be construed as a potential conflict of interest.
